# Prepandemic Predictors of Medication Adherence and HIV Viral Load During the First Year of COVID-19

**DOI:** 10.1097/QAI.0000000000003129

**Published:** 2022-11-07

**Authors:** Seth C. Kalichman, Lisa A. Eaton, Moira O. Kalichman, Soya S. Sam, Angela M. Caliendo

**Affiliations:** a Institute for Collaborative Health Intervention and Policy, University of Connecticut, Storrs, CT;; b Division of Infectious Diseases, The Miriam Hospital, Providence, RI; and; c Department of Medicine, Warren Alpert Medical School of Brown University, Providence, RI.

**Keywords:** HIV continuum of care, HIV treatment, young people living with HIV, COVID-19, COVID-19 impacts

## Abstract

Studies have reported significant immediate impacts of the COVID-19 pandemic on the social relationships and health care of people living with HIV. This study followed a closed cohort of young people living with HIV over the first year of the COVID-19 pandemic. Participants were men and women (N = 140) age 36 years and younger who were living with HIV and had demonstrated suboptimal adherence to antiretroviral therapy, unsuppressed HIV viral load, or active substance use in a run-in study. The results confirmed that participants continued to experience significant disruptions to their social relationships and health care over the course of the first year of the COVID-19 pandemic. There was evidence for sustained impacts on transportation, housing stability, and food security during the first year of COVID-19. Multivariable models showed that greater pre–COVID-19 social support predicted greater antiretroviral therapy adherence and greater HIV suppression (lower viral load) over the first year of the COVID-19 pandemic. Efforts to plan and prepare people living with HIV for future social crises, including future pandemics, should emphasize building and sustaining social support.

## INTRODUCTION

During the first year of the SARS-CoV-2 pandemic, before the availability of safe and effective vaccines, severe disease and death were most likely to occur among the elderly and people with chronic underlying health conditions, including people with compromised immune systems.^1–3^ For people living with HIV, unsuppressed virus is the hallmark of HIV disease progression, and unsuppressed HIV has been determined as a risk for increased COVID-19 severity.^4,5^ Because suboptimal adherence to antiretroviral therapies (ART) results in unsuppressed HIV, ART nonadherence and those factors that undermine ART adherence are therefore risk factors for increased vulnerability to COVID-19 in the absence of vaccination against SARS-CoV-2.^5,6^

Studies conducted in the first months of the COVID-19 pandemic also indicated that people living with HIV were being adversely affected by public health responses to COVID-19, particularly those requiring physical distancing. The most pressing concerns for people living with HIV were reports of limited access to health care, inability to access ART, and disruptions to ART adherence.^7^ Stay-at-home orders and public health campaigns intended to increase physical distancing exacerbated existing barriers to HIV care, such as limited access to transportation, strains on housing, and food insecurity.^8^ Furthermore, there were reports of early adverse impacts of the COVID-19 pandemic on people living with HIV with respect to discontinued social services and subsequent loss of social support leading to increased social isolation, psychological distress, and increased alcohol use.^9,10^

Much of what is known about the impacts of the COVID-19 pandemic on people living with HIV is based on rapidly conducted cross-sectional studies. As early as March 2020, the first month of the public health response to COVID-19, studies reported significant impacts on people living with HIV.^11^ For example, nearly all participants in a study conducted in the first few weeks of the pandemic had engaged in at least some degree of COVID-19 physical distancing and had limited their social contacts.^12^ Public health recommendations to reduce social contacts to avoid COVID-19 corresponded with health care disruptions.^12^ Physical distancing was also related to difficulty accessing food and medications and increased cancelation of health care appointments. However, ART adherence seemed to have improved during the initial month of the COVID-19 pandemic.^9^ Overall, studies in the early months of COVID-19 suggested that immediate mitigation efforts had the potential for prolonged impacts.

Here, we report a prospective cohort study of the social and health impacts of the COVID-19 pandemic experienced over the first year in a closed cohort of people living with HIV. Specifically, we conducted monthly phone interviews to assess participant reports of transportation, housing, food security, social support, and alcohol use, and collected objective measures of ART adherence and HIV viral load that were ongoing before March 2020. Measures to assess the impact of COVID-19 were also added to the study assessments. We hypothesized that participants with greater pre–COVID-19 challenges such as limited transportation, housing instability, food insecurity, lack of social support, and alcohol use would demonstrate persistent ART nonadherence and unsuppressed HIV viral load during the first year of COVID-19. We also examined the duration of physical distancing and interruptions to health care over the first year of the COVID-19 pandemic.

## METHODS

### Participant Recruitment

Participants were men and women living with HIV in the Atlanta metropolitan area. The sample was one of convenience accrued by advertising on social media outlets (eg, Facebook, 56% of the sample), study brochures placed in waiting areas of HIV clinical and social services (6%), and participants referring their social network members to this study (38%). Participants met the following inclusion criteria: (1) age between 19 and 36 years confirmed by showing a photograph identification, (2) HIV status confirmed by showing a name-matching HIV test result, HIV viral load report, or ART medication bottle; and (3) less than 85% adherent to ART on pill count assessments or viral unsuppressed determined by contemporaneous blood samples or not having an HIV care provider, or actively using substances determined by urine testing. Substance use was included as an entry criterion because of its close association with risks for treatment nonadherence.^13^

### First Year of COVID-19 Cohort

The current sample consists of the 140 active participants who were remaining in a larger study of people living with HIV (N = 435). Recruitment for the entire study occurred from September 2017 through December 2019 with participants in the current sample having been enrolled between August 2019 and December 2019. Starting in March 2020,^14^ measures of participant responses to COVID-19 were added to the ongoing monthly assessments. In addition, we extended the follow-up period from 15 months in the main study to 18 months to continue assessments for the purposes of this study. Data were aggregated into four 3-month periods, months 1–3, 4–6, 7–9, and 10–12 during the first year of COVID-19. For discrete variables (ie, detectable and undetectable viral load), any occurrence in the interval was coded as having occurred in that interval, and continuous variables were calculated as means for the period. The cohort was closed to new recruits, and the sample size reduced over time due to participants ending their study activities. Cohort size reduced from to 140 to 134 (95%) at 6 months, to 110 (79%) at 9 months, and 79 (56%) at 12 months.

### Procedures

After an enrollment appointment and informed consent, participants completed an interview which included a full accounting of their current ART regimen, blood sample collected for viral load testing, urine sample collected for substance use testing, and computerized self-administered surveys. All participants received counseling to assist with medication adherence. Participants were followed with monthly phone interviews that included unannounced pill counts to monitor ART adherence, and blood samples collected every 4 months for HIV viral load testing. Participants were monetarily compensated for each study activity completed, and the University of Connecticut Institutional Review Board approved the study.

### Measures

#### Participant Characteristics

Participants were asked their self-identified sex, race, age, years of education, income, and employment status.

### Social Impacts

During each monthly phone assessment, participants were asked about their housing stability, food security, alcohol use, and social support.

#### Housing Stability

We assessed current housing stability with a single item that read “I worried about having a place to stay” referring to the past month and participants responding Yes or No.

#### Food Security

To assess food insecurity, we used items adapted from the US Food Security Scale that have been validated and used by the US Census Bureau.^15^ Food security items were collected with respect to experiences accessing food in the past month and dichotomized as experienced or not experienced (see results for items).

#### Alcohol Use

The current use of alcohol was assessed with the 3-item consumption subscale of the Alcohol Use Disorders Identification Test [AUDIT-C,^16,17^]. The items assess frequency and quantity of alcohol use and binge drinking, alpha = 0.72.

#### Social Support

Social support was assessed with a 14-item scale tapping multiple aspects of social support.^18^ Example items include “I feel a strong emotional bond with at least one other person” and “There is someone I can turn to for advice about handling problems with my family.” Responses were 1 = “completely true” to 4 = “completely false,” with higher scores indicating greater social support alpha = 0.75.

### COVID-19 Physical Distancing and Impacts on Health care

Participants reported whether they had engaged in physical distancing to mitigate their risk for contracting COVID-19 and responded to questions regarding how COVID-19 affected their health care (see results for items). All of the physical distancing actions were recommended in the first year of COVID-19 by both the US CDC and the state of Georgia Department of Public Health. Each month participants reported whether they had engaged in the practices and whether they had experienced each impact during the time since their previous monthly phone assessment. Data were aggregated into four 3-month intervals.

### ART Adherence

We assessed ART adherence using phone-based unannounced pill counts. Unannounced pill counts are reliable and valid in assessing medication adherence when conducted in homes^19^ and on the phone.^20,21^ After an office-based training session that included a full accounting of all prescription medications, participants were called at unscheduled times over 25–30 day periods to calculate adherence.^22,23^ Monthly adherence, expressed as percent of pills taken as prescribed, was aggregated into 3-month intervals of the COVID-19 pandemic by taking the mean for the months included in each interval.

### HIV Viral Load

To determine HIV RNA plasma concentrations (viral load), participants provided 80 µL of fingerstick blood for dried blood spots collected in HemaSpot HF devices. Dried blood specimens during this study period were collected 3 times over a 12-month period using a remotely assisted (video chat) at-home self-collection procedure. Specimens were returned to the research office using next-day delivery and were frozen on receipt. HIV-1 viral load testing was conducted using the Abbott RealTi*m*e HIV-1 assay, a reverse transcription-PCR assay performed on the automated Abbott *m*2000 platform (Abbott Molecular Inc., Des Plaines, IL).^24^ Participants were given the option to provide a recent (within 1-month of assessment date) viral load test result obtained from a health care provider. Detectable/undetectable viral load was defined as 1000 copies/mL, a threshold that nearly eliminates errors caused by viral load blips or assay variability.^25^

### Data Analyses

We conducted descriptive analyses for participants by partitioning the sample based on pre–COVID-19 undetectable and detectable viral load. For descriptive analyses, we performed contingency table χ^2^ tests for categorical variables and independent *t*-tests for continuous measures. We also examined physical distancing and COVID-19 health care disruptions for undetectable and detectable viral load groups within each period by performing contingency table χ^2^ tests.

For the main study analyses, regression models tested predictors of ART adherence and HIV viral load during the first year of COVID-19. For ART adherence, we first examined predictors of pre–COVID-19 ART adherence to establish pre–COVID-19 associations in the unadjusted and adjusted models. Next, we tested the predictors of adherence during four 3-month intervals adjusted for all variables in the models. We present model coefficients (regression weights and standard errors) and model effects with R^2^ values.

For HIV viral load, we first tested pre–COVID-19 predictors of pre–COVID-19 HIV viral load in unadjusted and adjusted models. Next, we conducted prospective logistic regressions with pre–COVID-19 predictors of HIV viral load during the first year of COVID-19. We present the bivariate models adjusted for pre–COVID-19 viral load and the multivariable model predicting viral load during the first year of COVID-19 with all pre–COVID-19 predictors included in the model. The models therefore report unadjusted and adjusted odds ratios with 95% confidence intervals. All statistical tests use all available data, and we defined significance as *P* < 0.05.

## RESULTS

Table 1 summarizes the demographic characteristics for participants partitioned by pre–COVID-19 undetectable and detectable HIV viral loads. Most participants were African American men with annual incomes under $30,000. Nearly two-thirds of participants (62%) had been tested for COVID-19 over the first year of the COVID-19 pandemic, and 17% had received a positive COVID-19 test result. There were no differences between participants with pre–COVID-19 undetectable and detectable viral loads for any participant characteristics.

**TABLE 1.** Participant Characteristics Partitioned by Pre–COVID-19 Pandemic Undetectable Vs Detectable HIV Viral Load

	Undetectable		Detectable		
	Viral Load (N = 97)		Viral Load (N = 43)		
	N	%	N	%	χ^2^
Cisgender man	69	71	34	80	3.7
Transgender man	1	1	1	2	
Cisgender woman	17	17	4	9	
Transgender woman	10	11	4	9	
African American	87	90	41	95	5.9
Annual income < $30,000	79	81	38	88	2.2
Currently employed	20	21	10	23	2.2
Has been tested for COVID-19	56	58	31	72	2.6
Received positive COVID-19 test result	15	16	9	21	0.6
	M	SD	M	SD	t
Age	29.3	3.4	28.8	4.2	0.7
Years of education	13.5	1.3	13.3	1.6	0.6

### COVID-19 Social Distancing and Health care Disruptions

Table 2 summarizes the percent of participants reporting social distancing responses and disruptions in health care due to pandemic responses over the first year of COVID-19 partitioned by pre–COVID-19 undetectable and detectable viral load. Greater than 90% of participants indicated staying indoors and away from public places at all time points over the first year of COVID-19. A sizable majority of participants, over 60% at all time points, also reported cancelling plans with others and asking others to stay away in an effort to avoid contracting COVID-19. It was also common for participants to report COVID-19–related disruptions to their health care, with more than one of 3 participants missing health care appointments due to COVID-19 across time intervals. During the first 6 months of COVID-19, 2 of 3 participants indicated that their health care providers cancelled a medical appointment, whereas cancellations occurred for only one of 3 participants during months 7%–9% and 16% in the final 3 months of the first year. Disruptions in accessing ART occurred for nearly one in 5 participants across the first year of the pandemic. Comparisons of participants with pre–COVID-19 undetectable and detectable viral loads at each period did not show any significant group differences in social distancing and health care disruptions.

**TABLE 2.** Percent of Participants With Pre–COVID-19 Pandemic Undetectable and Detectable HIV Viral Loads Reporting Actions to Physically Distance and Disruptions in Health care Over the First Year of the COVID-19 Pandemic

	Months into the First Year of COVID-19			
	Months 1–3 (N = 140)	Months 4–6 (N = 134)	Months 7–9 (N = 110)	Months 10–12 (N = 89)
Staying indoors and away from public places				
Undetectable viral load	89	92	92	94
Detectable viral load	95	94	95	96
Canceled plans that involve other people.				
Undetectable viral load	76	82	75	70
Detectable viral load	74	84	70	75
Asking others to stay away to avoid getting the COVID-19				
Undetectable viral load	68	73	65	61
Detectable viral load	76	77	70	69
Missed a health care appointment due to COVID-19				
Undetectable viral load	39	40	37	32
Detectable viral load	41	25	36	45
Provider cancelled a care appointment due to COVID-19				
Undetectable viral load	62	68	35	15
Detectable viral load	65	62	42	17
Ran out of ART due to COVID-19				
Undetectable viral load	17	21	29	13
Detectable viral load	18	28	25	24

No significant differences between groups at each period.

### Transportation, Housing, and Food Insecurity

It was common for participants to experience a lack of transportation, unstable housing, and food insecurity pre–COVID-19 and across the first year of COVID-19. Table 3 summarizes the percent of participants with pre–COVID-19 undetectable and detectable HIV viral loads who experienced a lack of transportation, unstable housing, and food insecurity over the first year of COVID-19. Within each time interval, participants with pre–COVID-19 detectable HIV viral loads experienced more transportation difficulty, unstable housing, and food insecurity than their counterparts with undetectable viral loads. The differences between viral load groups was significant for lacking transportation in the first 3 months of COVID-19 and for being unable to get transportation to health care providers, unstable housing, and running out of food during the 7–9 months interval.

**TABLE 3.** Percent of Participants With Pre–COVID-19 Pandemic Undetectable and Detectable HIV Viral Loads Experiencing Lack of Transportation, Unstable Housing, Food Insecurity, ART Adherence, Social Support, and Alcohol Use Over the First Year of the COVID-19 Pandemic

		Months into the First Year of COVID-19			
	Pre–COVID-19 (N = 140)	Months 1–3 (N = 140)	Months 4–6 (N = 134)	Months 7–9 (N = 110)	Months 10–12 (N = 89)
Unable to access transportation					
Undetectable viral load	55	47*	41	41	31
Detectable viral load	65	71	46	70	62
Unable to get transportation to a health care appointment					
Undetectable viral load	38	63	25	33*	29
Detectable viral load	47	51	34	58	52
Unstable housing					
Undetectable viral load	46*	38	28	38*	40
Detectable viral load	60	57	32	65	45
Food insecurity					
Ran out of food and could not buy more					
Undetectable viral load	70	56	44	41*	46
Detectable viral load	67	62	54	66	47
Food did not last and reduced meals					
Undetectable viral load	66	57	45	46	49
Detectable viral load	76	71	54	66	52
Was hungry and could not afford food					
Undetectable viral load	58	48	35	36	35
Detectable viral load	59	57	48	65	47

	Months into the First Year of COVID-19									
	Pre–COVID-19		Months 1–3		Months 4–6		Months 7–9		Months 10–12	
	M	SD	M	SD	M	SD	M	SD	M	SD
ART adherence										
Undetectable viral load	65.7	25.4	71.0*	25.5	67.6*	23.7	66.4*	26.8	70.0*	22.2
Detectable viral load	58.6	29.4	57.7	32.9	55.6	29.4	58.7	32.6	54.6†	29.4
Social support										
Undetectable viral load	3.3*	0.4	3.1†	0.8	3.0†	0.6	3.0*†	0.6	3.0†	0.6
Detectable viral load	3.2	0.3	2.8†	0.6	2.9†	0.7	2.5†	0.6	2.8†	0.6
Alcohol consumption score (AUDIT-C)										
Undetectable viral load	3.0	2.4	4.3	5.1	2.4*	2.4	2.6*	2.2	2.1	2.6
Detectable viral load	2.7	2.5	3.2	4.9	1.4†	1.6	1.4†	1.5	1.8	2.4

* Significant difference between viral load groups, *P* < 0.05.

† Significant differences within groups for time intervals compared with pre–COVID-19, *P* < 0.05.

### Adherence, Social Support, and Alcohol Use During the First Year of COVID-19

Table 3 summarizes the means and standard deviations for ART adherence, social support, and alcohol use among participants with undetectable and detectable viral loads over the first year of COVID-19. Group comparisons within each period showed that participants with pre–COVID-19 undetectable viral loads persisted in having higher adherence than their counterparts with detectable HIV viral loads at every period. Within-group comparisons showed that participants with pre–COVID-19 detectable HIV viral loads had lower adherence during the 9–12 month interval relative to their pre–COVID-19 adherence. For social support, both viral load groups demonstrated diminished social support throughout the first year of COVID-19, with the detectable viral load group indicating less social support than the undetectable viral load group during months 4–6. Finally, regarding alcohol use, participants with undetectable viral loads reported greater alcohol use during months 4–6, while the participants with pre–COVID-19 detectable viral loads indicated significant reductions in alcohol use during both the 4–6 month and 7–9 month intervals.

### Predictors of ART Adherence

The results indicated that higher social support was the only pre–COVID-19 predictor associated with higher pre–COVID-19 ART adherence in the unadjusted models (see Table 4). However, there were no significant predictors of pre–COVID-19 adherence in the adjusted model. In the prospective multivariable analyses, older age and higher pre–COVID-19 social support predicted greater ART adherence during the first 3-months of COVID-19. In addition, higher social support remained the only significant predictor of greater ART adherence over the next 4–6 month and 7–9 month intervals. Finally, the results indicated that lacking transportation to health care during the pre-COVID assessment predicted lower ART adherence in months 9–12.

**TABLE 4.** Predictors of ART Adherence During the First Year of COVID-19 From Pre–COVID-19 Lack of Transportation, Unstable Housing, Food Insecurity, Social Support, and Alcohol Use

Pre–COVID-19 Predictor Variable	Unadjusted Predictors of Pre–COVID-19 Adherence		Adjusted Predictors of Pre–COVID-19 Adherence		Adjusted Predictors of Adherence During COVID-19 Months 1–3		Adjusted Predictors of Adherence During COVID-19 Months 4–6		Adjusted Predictors of Adherence During COVID-19 Months 7–9		Adjusted Predictors of Adherence During COVID-19 Months 10–12	
	B	Se	B	se	B	se	B	se	B	se	B	se
Participant age	0.003	0.006	0.006	0.006	0.012*	0.006	0.005	0.006	0.010	0.008	0.015	0.009
Lack of transportation to health care	−0.043	0.033	−0.035	0.052	−0.057	0.039	−0.013	0.038	−0.048	0.046	−0.120*	0.051
Unstable housing	−0.045	0.028	−0.006	0.054	0.001	0.035	−0.010	0.035	−0.013	0.042	−0.004	0.049
Food insecurity	−0.015	0.011	−0.005	0.015	0.008	0.015	0.010	0.015	0.010	0.018	0.006	0.020
Social support	0.119*	0.054	0.100	0.062	0.174**	0.063	0.144*	0.063	0.168*	0.078	0.086	0.083
Alcohol consumption	0.012	0.009	0.013	0.009	0.018	0.009	0.007	0.009	−0.001	0.011	−0.005	0.013
Model			F = 1.46, R^2^ = 0.06		F = 3.4**, R^2^ = 0.13		F = 1.2, R^2^ = 0.05		F = 1.5, R^2^ = 0.08		F = 1.7, R^2^ = 0.11	

Two-tailed tests *P < 0.05. **P < 0.01.

**TABLE 5.** Predictors of Detectable HIV Viral Load During the First Year of COVID-19 From Pre–pandemic Lack of Transportation, Housing Instability, Food Insecurity, Social Support, and Alcohol Use

Pre–COVID-19 Predictor Variable	Unadjusted Predictors of Pre–COVID-19 Detectable Viral Load		Adjusted Predictors of Pre–COVID-19 Detectable Viral Load		Unadjusted Predictors of Detectable Viral Load During the First Year of COVID-19		Adjusted Predictors of Detectable Viral Load During the First Year of COVID-19	
	OR	95% CI	OR	95% CI	OR	95% CI	OR	95% CI
Pre–COVID-19 viral load					4.09**	1.84 to 9.06	3.70**	1.49 to 9.16
Participant age	0.96	0.87 to 1.06	0.96	0.86 to 1.06	1.09	0.97 to 1.21	1.08	0.96 to 1.22
Lack of transportation to health care	1.77*	1.03 to 3.04	2.29	0.96 to 5.46	2.41**	1.36 to 4.27	1.69	0.85 to 3.36
Unstable housing	1.65*	1.05 to 2.6	1.33	0.55 to 3.23	1.58	0.99 to 2.53	1.29	0.68 to 2.44
Food insecurity	1.19	0.98 to 1.44	1.05	0.82 to 1.34	1.16	0.95 to 1.41	0.87	0.65 to 1.17
Social support	0.44	0.18 to 1.09	0.71	0.24 to 2.09	0.12**	0.04 to 0.35	0.16**	0.04 to 0.56
Alcohol consumption	0.94	0.81 to 1.10	0.39	0.80 to 1.09	0.94	0.80 to 1.10	0.94	0.79 to 1.12
Model			χ^2^ = 10.42 Cox & Snell R^2^ = 0.07				χ^2^ = 32.49**, Cox & Snell R^2^ = 0.21	

Two-tailed tests *P < 0.05; **P < 0.01.

### Predictors of HIV Viral Load

We first examined the associations of pre–COVID-19 predictors with pre–COVID viral load (see Table 5). The results showed that lack of transportation to health care providers and unstable housing were associated with pre–COVID-19 detectable viral loads in the unadjusted models. However, there were no significant predictors of pre–COVID-19 viral load in the adjusted model. In the prospective models that included pre–COVID-19 viral load as a control variable, pre–COVID-19 lack of transportation to health care providers, and less social support were associated with detectable viral loads during the first year of COVID-19 in the unadjusted models. In the prospective model adjusting for all variables including pre–COVID-19 viral load, lower prepandemic social support predicted detectable HIV viral load during the first year of the COVID-19 pandemic.

## DISCUSSION

Substantial personal and social impacts of COVID-19 on people living with HIV were widely reported in the first weeks of the COVID-19 pandemic and persisted over the first year. We found that people living with HIV in our cohort continued to stay home, avoided public places, and cancelled plans with others to avoid contracting COVID-19. In addition, we observed sustained interruptions to health care regarding participants cancelling appointments and experiencing gaps in their ART prescriptions. However, there was a decline in providers canceling appointments after the first 6 months of COVID-19, with 75% fewer participants report provider cancelations in the final quarter of the first year compared with the first 6 months. Furthermore, challenges posed to lack of transportation, housing instability, and food insecurity persisted throughout the first year of COVID-19. Although we observed significant adverse impacts throughout the cohort, there was a clear pattern of greater impacts for participants who had pre–COVID-19 detectable HIV viral loads. By contrast, we observed transient reductions in alcohol use among participants who had pre–COVID-19 detectable viral loads, possibly an artifact of diminished personal resources that are encompassed by social support.

Although some early reports suggested that the COVID-19 pandemic may have had a positive impact on some health behaviors among people living with HIV, such as improved ART adherence,^12,26,27^ we found those effects were short-lived. ART adherence remained greater, albeit suboptimal as per our inclusion criteria, for participants who had a pre–COVID-19 undetectable viral load compared with those with detectable viral loads. In the prospective analyses, we found that older age was associated with improved adherence in the first months of COVID-19 and lack of pre–COVID-19 access to transportation predicted lower adherence in the final quarter of the first year.

Participants generally demonstrated significant reductions in social support over the first year of COVID-19. Pre–COVID-19 social support, however, was a robust predictor of ART adherence, with greater social support predicting greater adherence over the first 9 months of COVID-19. These results were echoed in prospective analyses of HIV viral load, with greater pre–COVID-19 social support predicting greater viral suppression over the first year of COVID-19. Social support therefore may have contributed to a resilience in dealing with COVID-19 and the challenges people living with HIV faced in managing their health care during that difficult time.^27^

These study findings should be interpreted in light of their methodological limitations. The sample for this study was one of convenience and cannot be considered representative of people living with HIV. The sample was also largely male participants and African American and therefore limited in its generalizability. In addition, analyses conducted during the final months of the year had less statistical power than analyses conducted pre–COVID-19 and in the early months of the pandemic. Our measure of social support was also limited by not consisting of reliable subscales for emotional and tangible support. With these limitations in mind, our findings have implications for managing HIV infection during pandemics and other unforeseen social crises.

The importance of social support in optimizing and sustaining the health care and health of people living with HIV was apparent in the current findings. Beyond pre–COVID-19 adherence and viral load, only social support demonstrated a robust relationship with ART adherence and suppressed HIV replication over the first year of COVID-19. Social support is a well-known stress buffer^28–30^ and is established as a key factor in sustained ART adherence.^31–33^ Early reports in the COVID-19 pandemic suggested that social support was playing an important role in retaining people living with HIV in health care,^34^ and our findings show that these benefits persisted over the course of the first year. In addition, previous research has shown similar effects of social support for people living with HIV through other social crises, natural disasters, and traumatic life events,^35^ such as earthquakes^36^ and hurricanes.^37,38^ Efforts to build and maintain social support among people living with HIV may be a critical feature for future crisis and pandemic preparedness.
